# Direct Regulation of Hyperpolarization-Activated Cyclic-Nucleotide Gated (HCN1) Channels by Cannabinoids

**DOI:** 10.3389/fnmol.2022.848540

**Published:** 2022-04-06

**Authors:** Sultan Mayar, Mina Memarpoor-Yazdi, Ahmad Makky, Romina Eslami Sarokhalil, Nazzareno D'Avanzo

**Affiliations:** Département de Pharmacologie et Physiologie, Université de Montréal, Montréal, QC, Canada

**Keywords:** HCN channel, cannabinoids, cannabidiol, Δ9-THC, ion channel

## Abstract

Cannabinoids are a broad class of molecules that act primarily on neurons, affecting pain sensation, appetite, mood, learning, and memory. In addition to interacting with specific cannabinoid receptors (CBRs), cannabinoids can directly modulate the function of various ion channels. Here, we examine whether cannabidiol (CBD) and Δ9-tetrahydrocannabinol (THC), the most prevalent phytocannabinoids in *Cannabis sativa*, can regulate the function of hyperpolarization-activated cyclic-nucleotide-gated (HCN1) channels independently of CBRs. HCN1 channels were expressed in *Xenopus* oocytes since they do not express CBRs, and the effects of cannabinoid treatment on HCN1 currents were examined by a two-electrode voltage clamp. We observe opposing effects of CBD and THC on HCN1 current, with CBD acting to stimulate HCN1 function, while THC inhibited current. These effects persist in HCN1 channels lacking the cyclic-nucleotide binding domain (HCN1ΔCNBD). However, changes to membrane fluidity, examined by treating cells with TX-100, inhibited HCN1 current had more pronounced effects on the voltage-dependence and kinetics of activation than THC, suggesting this is not the primary mechanism of HCN1 regulation by cannabinoids. Our findings may contribute to the overall understanding of how cannabinoids may act as promising therapeutic molecules for the treatment of several neurological disorders in which HCN function is disturbed.

## Introduction

Hyperpolarization-activated cyclic-nucleotide-gated (HCN) channels are widely expressed in the central and peripheral nervous systems. All four isoforms (HCN1-4) are expressed in the brain (Pape, 1996; Santoro et al., 1997, 1998; Ludwig et al., 1998; Moosmang et al., 2001) where they play a role in setting the resting membrane potential, dendritic integration, neuronal pacemaking, and establishing the action potential threshold (Pape, 1996). HCN channels are important for learning and memory, pain sensation, sour taste sensation, and vision. HCN1^−/−^ mice show impaired motor learning but enhanced spatial learning and memory (Nolan et al., 2003, 2004) and enhanced susceptibility to kainic acid-induced seizures (Huang et al., 2009). HCN2^−/−^ mice presented symptoms of absence epilepsy and tremoring (Ludwig et al., 2003). Gain and loss of function mutations in HCN1 and HCN2 have also been identified in patients with various forms of epilepsy (Tang et al., 2008; Dibbens et al., 2010; DiFrancesco et al., 2011; Nakamura et al., 2013; Nava et al., 2014). Altered HCN-cAMP signaling in prefrontal cortex networks also appears to contribute to the working memory deficits in schizophrenia and stress (Arnsten, 2011; Paspalas et al., 2013; Gamo et al., 2015). HCN channels are also highly expressed in primary afferent (sensory) neurons such as the dorsal root ganglion (Scroggs et al., 1994; Villiere and McLachlan, 1996; Yagi and Sumino, 1998; Cardenas et al., 1999; Abdulla and Smith, 2001; Chaplan et al., 2003; Yao et al., 2003; Tu et al., 2004; Masuda et al., 2006; Onoda et al., 2006) with HCN1 and HCN2 predominant in large and small-sized neurons, respectively (Moosmang et al., 2001; Chaplan et al., 2003; Tu et al., 2004). HCN2^−/−^ mice do not demonstrate neuropathic pain in response to mechanical or thermal stimuli (Emery et al., 2011) suggesting that I_h_ drives action potential firing to initiate neuropathic pain in nociceptors. Mutations in the scaffolding protein SHANK3 may predispose people to autism by inducing an I_h_ channelopathy with increased neuronal input resistance, enhanced neuronal excitability, and reduced synaptic transmission (Yi et al., 2016).

Cannabinoids are a broad class of molecules that act primarily on neurons, affecting pain sensation, appetite, mood, learning, and memory. There are 3 classes of cannabinoids: (1) endocannabinoids produced naturally by the body, (2) phytocannabinoids from some plants, and (3) synthetic cannabinoids. Cannabinoids may provide effective treatments for addiction, pain, epilepsy, major mood disorders, anxiety, post-traumatic stress disorder, spasticity in multiple sclerosis, Parkinson's disease, and Huntington's disease (Cunha et al., 1980; Hill et al., 2012; The Health Effects of Cannabis Cannabinoids, 2017; Russo, 2018). However, to use them therapeutically, without negative side effects, we must understand exactly how they interact with their targets to affect nerve function.

**Figure 1:**
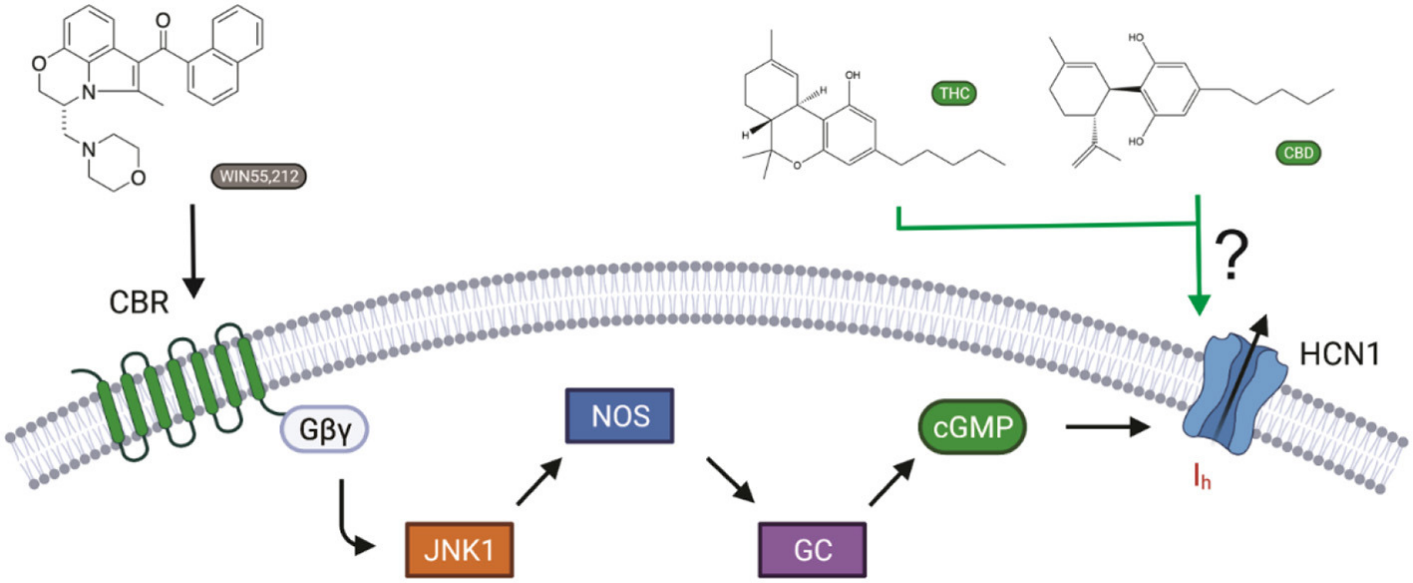
Cannabinoid regulation of I_h_ in neurons. The synthetic cannabinoid WIN55,212-2 affects memory *via* increasing I_h_ by activating CB1 receptors, leading to JNK-mediated increases in activated nitric oxide synthase (NOS), resulting in increased guanylyl cyclase activity and, in turn, cGMP (Hill et al., 2012). However, cannabinoids have also been shown to modify the function of several ion channels independently of cannabinoid receptors (CBRs) activation. Here, we ask if hyperpolarization-activated cyclic-nucleotide-gated (HCN1) channels can be directly modulated by cannabidiol (CBD) and Δ9-tetrahydrocannabinol (THC) (Maroso et al., 2016).

Cannabinoids bind to cannabinoid receptors (CB1R or CB2R). However, activating CBRs cannot directly alter electrical excitability in neurons, since CBRs do not generate electrical signals on their own. Instead, membrane potential and electrical signaling in all excitable cells, including neurons, are generated by ion channels embedded in the cell membrane. Recently, it has been shown that the synthetic cannabinoid WIN55,212-2 affects memory by activating CB1 receptors, leading to intracellular signaling changes that affect the I_h_ current generated by hyperpolarization-activated HCN channels (Maroso et al., 2016) (Figure 1). The CB1R–I_h_ pathway does not involve adenylyl cyclase/cAMP formation, since CB1Rs decrease cAMP levels through Gα_i_. Instead, CB1R activation involves JNK-mediated increases in activated nitric oxide synthase (NOS), resulting in increased guanylyl cyclase activity and, in turn, cGMP.

In addition to activating CBRs, cannabinoids can also directly bind ion channels, or affect their function by altering the physiochemical properties of the cell membrane. WIN 55,212–2, Δ^9^-tetrahydrocannabinol (THC) and cannabidiol (CBD) alter the voltage-dependence of activation and inactivation in several Nav channels (Okada et al., 2005; Ghovanloo et al., 2018). THC and CBD also inhibit T-type calcium channels (Ross et al., 2008). Some TRP channel subfamilies, including TRPV1, are activated and desensitized by CBD, while TRPM8 is inhibited (De Petrocellis et al., 2011, 2012; Iannotti et al., 2014). On the other hand, anandamide, THC, and CBD potentiate homomeric and heteromeric glycine receptors (Hejazi et al., 2006; Ahrens et al., 2009). Given the important role of HCNs in synaptic integration, mood, pain sensation, and memory, and the knowledge that cannabinoids may have therapeutic potential for these same disorders and can directly regulate the function of several ion channels, we aimed to determine if cannabinoids have direct effects on HCN channels that are independent CBR activation.

## Materials and Methods

### Drugs and Reagents

Drugs, cannabidiol (CBD), and Δ9-THC (Sigma-Aldrich, USA) were prediluted in 99.8% methanol at a concentration of 1.0 mg/ml. Detergent, Triton™ X-100 (Sigma-Aldrich, USA) was diluted to a working concentration of 10 mM with distilled water from a stock solution.

### Molecular Biology and Cell Expression

cDNA coding for the mouse HCN1 gene was previously subcloned into expression vector pGH19 (provided by Dr. William N. Zagotta, University of Washington, Seattle, Washington). The mouse HCN1-CX5 construct (denoted as HCN1-ΔCNBD in this article) was previously subcloned into expression vector pGH19 (provided by Bina Santoro, Columbia University, New York). To obtain RNA, NheI (New England Biolabs) was used to linearize both cDNA constructs of mHCN1 and ~1.0 μg of linearized cDNA was used for *in vitro* transcription synthesis using the mMESSAGE mMACHINE™ T7 Transcription kit (Thermo Fisher Scientific, Life Technologies, USA).

All the experiments were performed using unfertilized *Xenopus* oocytes, extracted from anesthetized female *Xenopus laevis*. Once extracted, oocytes were injected with 4.6 ng of mHCN1 using a Drummond Nanoject II injector (Drummond Scientific Company). Prior to injection, oocytes were subject to a controlled temperature of 17–19°C and placed in vials containing Barth antibiotic solution (mM): 90 NaCl, 3 KCl, 0.82 MgSO_4_.7H_2_O, 0.41 CaCl_2_.2H_2_O, 0.33 Ca(NO_3_)_2_.4H_2_O, and 5 HEPES supplemented with 100 U/ml of penicillin-streptomycin and 10 mg/ml of kanamycin stock (10 mg/ml). Post injection cells were incubated in Barth antibiotic serum solution supplemented with ~5% horse serum. Cells were expressed and ready to be used in electrophysiological recordings 1–3 days post injection.

### Electrophysiological Recordings

Electrophysiological studies were conducted using the two-electrode voltage clamp (TEVC) technique. Borosilicate rapid fill microelectrode pipettes (1.0 mm OD ×0.5 mm ID/Fiber from FHC Inc., USA) were filled with filtered 1 M KCL solution. Oocytes expressing wild-type HCN1 and HCN1-ΔCNBD were recorded in a bath solution containing (in mM) 89 KCl, 15 HEPES, 0.4 CaCl_2_, and 0.8 MgCl_2_, pH = 7.4. Macroscopic currents were recorded using Oocyte Voltage Clamp (OC-725C) amplifier (Warner Instruments, USA) and digitized using the Digidata 1322A data acquisition apparatus (Molecular Devices). All the data were acquired using the software Clampex 10.5 at a sampling rate of 5 KHz with a filter of 1 KHz. Repetitive pulse protocols involved 2 s pulses to −130 mV hyperpolarized voltage every 30 s from a holding potential (V_H_) of 0 mV. HCN1 activation was assessed by 3.5 s test-steps between −160 and −30 mV (ΔV = +10 mV) from a V_H_ = 0 mV, followed by a 3 s step to −160 mV. Deactivation was assessed by applying a 1.75 s prepulse to −130 mV, followed by test pulses from +50 to −70 mV (ΔV = −10 mV). Hysteresis was also monitored by applying voltage ramps from 0 to −150 mV and back to 0 mV at varying speeds. In all recordings, cells were held at the holding potential for an inter-pulse time of 27 s to allow the channels to fully recover between sweeps. Cannabinoids were added to the bath solution in 10 μM increments only after recordings of the previous condition had stabilized (i.e., current density remained unchanged between recordings at that concentration; typically, 15–30 min after their addition). Equimolar quantities of methanol used to solvate the cannabinoids to their listed concentrations were used as controls. Therefore, where presented, MeOH (X μM) represents the quantity of methanol used to dilute CBD or THC to X μM, and not X μM of methanol. All recordings were conducted at room temperature.

### Data Analysis and Statistics

All recordings were analyzed offline using the Clampfit (Molecular Devices) software. Data were analyzed and plotted using Origin 8.0 software (Northampton, MA, USA) or GraphPad Prism (Version 8.1.1, San Diego, CA). Current-voltage (I–V) relationships were analyzed using built-in software in pClamp, taking each respective voltage to an inquired current. The I–V relationship was fit with the Boltzman I–V equation:


(1) $$\begin{array}{l} I\;=\;\frac{\left(V_{m}\;-\;V_{rev}\right)g_{\max}}{1\;+\;e^{\frac{V_{m}\;-\;V_{\frac{1}{2}}}{k}}} \end{array}$$

Activation and deactivation kinetics were determined with mono-exponential fits of test pulses after the initial lag period. Steady-state activation curves were fit with a standard Boltzmann equation:


(2) $$\begin{array}{l} \frac{G}{G_{Max}}\;=\;\frac{1}{1\;+\;e^{\frac{V_{m}\;-\;V_{\frac{1}{2}}}{k}}} \end{array}$$

where V_m_ corresponds to the test pulse, V_1/2_ is the midpoint of activation, and k is the slope factor. EC_50_/IC_50_ values were determined by fitting concentration dependence curves with the Hill equation:


(3) $$\begin{array}{l} \frac{I}{I_{0}}\;=\;\frac{1}{1\;+\;{\left(\frac{EC_{50}\;or\;IC_{50}}{\left[A\right]}\right)}^{n}}, \end{array}$$

where I_0_ is the baseline current for HCN1 prior to treatment.

Data are presented as means (±) standard error. Statistical significance for I–V curves was measured using two-way ANOVA with Tukey HSD *post-hoc* analysis. V_1/2_'s of steady-state dependencies were determined for each recording and pooled for a given treatment then analyzed by one-way ANOVA with Tukey *post-hoc* analysis. Mean activation and deactivation kinetics (from −20 to −70 mV) were analyzed using the Zar method for significance (Zar, 1984).

## Results

**Figure 2:**
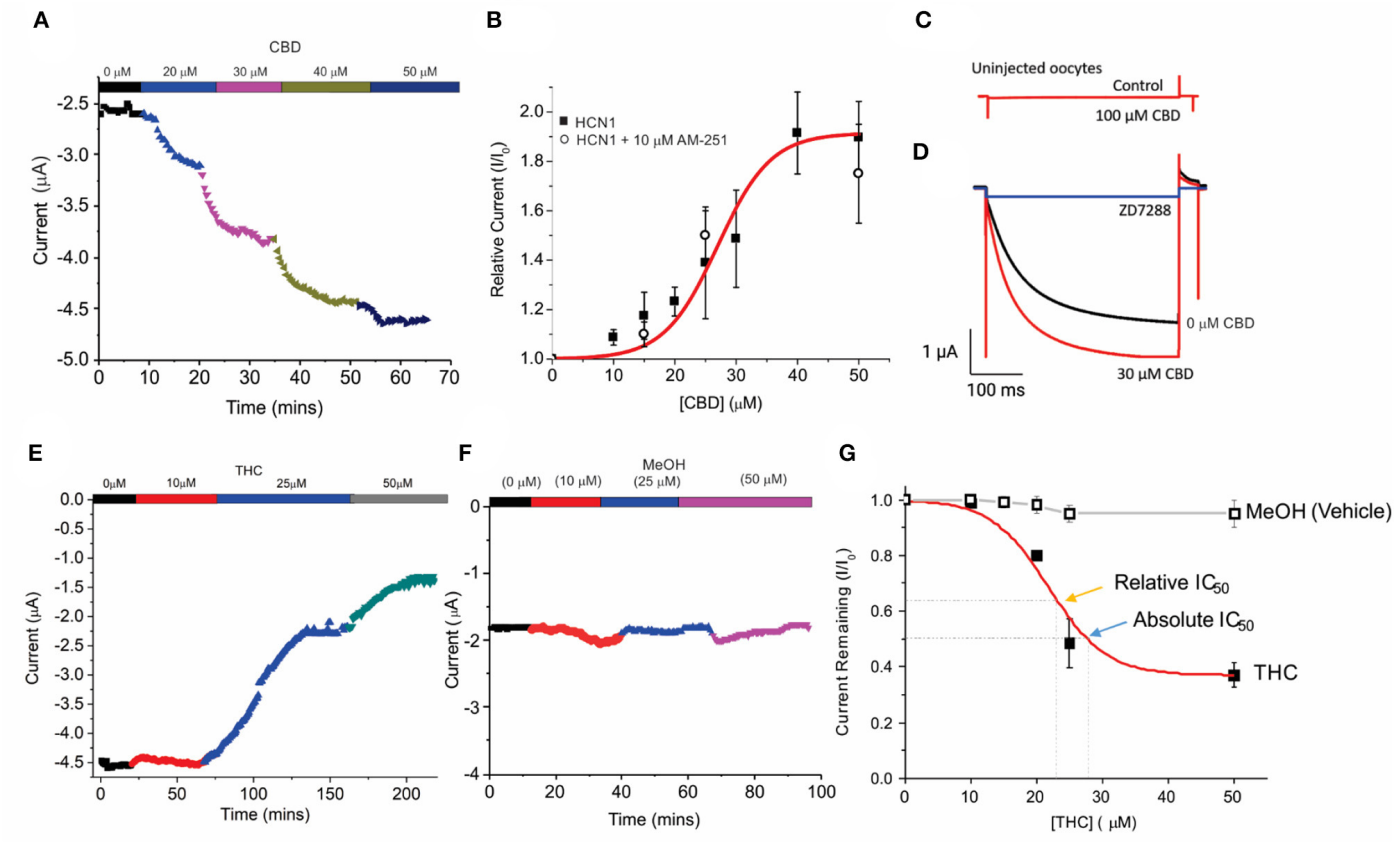
Concentration-dependent regulation of HCN1 current by THC and CBD. **(A)** Steady-state current values from a representative cell following repetitive pulses to −130 mV from V_H_ = 0 mV with the addition of 0, 10, 20, 30, and 50 μM CBD. **(B)** Concentration dependence of HCN1 activation by CBD (■) at −130 mV (*n* = 13). This activation of HCN1 persists in the presence of the CB1R antagonist 10 μM AM-251 (°) (*n* = 5). A total of 50% Max response (EC_50_) for CBD is elicited at 28.5 μM, with a 91% maximal increase in current. **(C)** CBD has no effects on uninjected oocytes, indicating that we are not observing effects on background currents. **(D)** CBD activated currents can be fully inhibited by 500 μM ZD7288. This indicates that CBD is only activating HCN1 currents, and not activating a background current in oocytes. **(E)** Steady-state currents from a representative cell following repetitive pulses to −130 mV in 0, 10, 25, and 50 μM THC. **(F)** Steady-state currents from a representative cell following repetitive pulses to −130 mV in the presence of the quantity of methanol used as a vehicle for 0, 10, 25, and 50 μM CBD/THC. **(G)** Concentration dependence of HCN1 inhibition by THC (■) or methanol (□) at −130 mV (*n* = 5). THC induces a 63% maximal block of HCN1 currents, with a half-maximal response (Relative IC_50_) of 21.8 μM. 50% block of total current (Absolute IC_50_) occurs at 28.9 μM. Methanol induces < a 5% decrease in current at concentrations above 20 μM (*n* = 4).

Unfertilized *Xenopus oocytes* lack CBRs (Xenbase.org) (Karimi et al., 2018) and thus, provide an ideal system to examine the direct effects of cannabinoids on HCN channels by two-electrode voltage-clamp (TEVC). Using a repetitive pulse protocol to hyperpolarized potentials (−130 mV from a holding potential V_H_ = 0 mV), we assessed the effects of adding CBD or THC to the bath solution at increasing concentrations (Figure 1). To ensure the effects of CBD and THC could be differentiated from the vehicle (methanol), we first examined the effects of equimolar quantities of methanol used to solvate the cannabinoids to their listed concentrations. We saw a negligible change (<5%) in overall current over the course of nearly 2 h at varying concentrations (Figures 2F,G). We observed a concentration-dependent increase in HCN1 current with the addition of CBD, which can then be inhibited by a saturating concentration of ZD7288 (Figures 2A,C,D). At −130 mV, the concentration dependence of CBD activation of HCN1 shows 50% max response (EC_50_) at 28.5 μM (slope = 0.1) with up to 91% maximal increase in current (Figure 2B). This effect is observed even in the presence of the CB1 receptor antagonist AM-251 (Figure 2B), providing further support that CBRs are not functionally expressed in *Xenopus* oocytes. Furthermore, the addition of CBD to uninjected oocytes does not stimulate the activation of a “background” current (Figure 2C), indicating that the observed changes are only due to the effect on HCN1 currents.

We then studied the effect of Δ^9^- THC on HCN1. Contrary to the effect of CBD, THC has an inhibitory effect on HCN1 (Figure 2E). Using repetitive pulses to −130 mV, we observe a concentration-dependent inhibition by THC with a maximal inhibitory response of THC of 63% (Figure 2G) and a half-maximal response (relative IC_50_) at 21.8 μM (slope = −0.1). A total of 50% block of HCN1 current by THC (absolute IC_50_) occurs at 28.9 μM.

**Figure 3:**
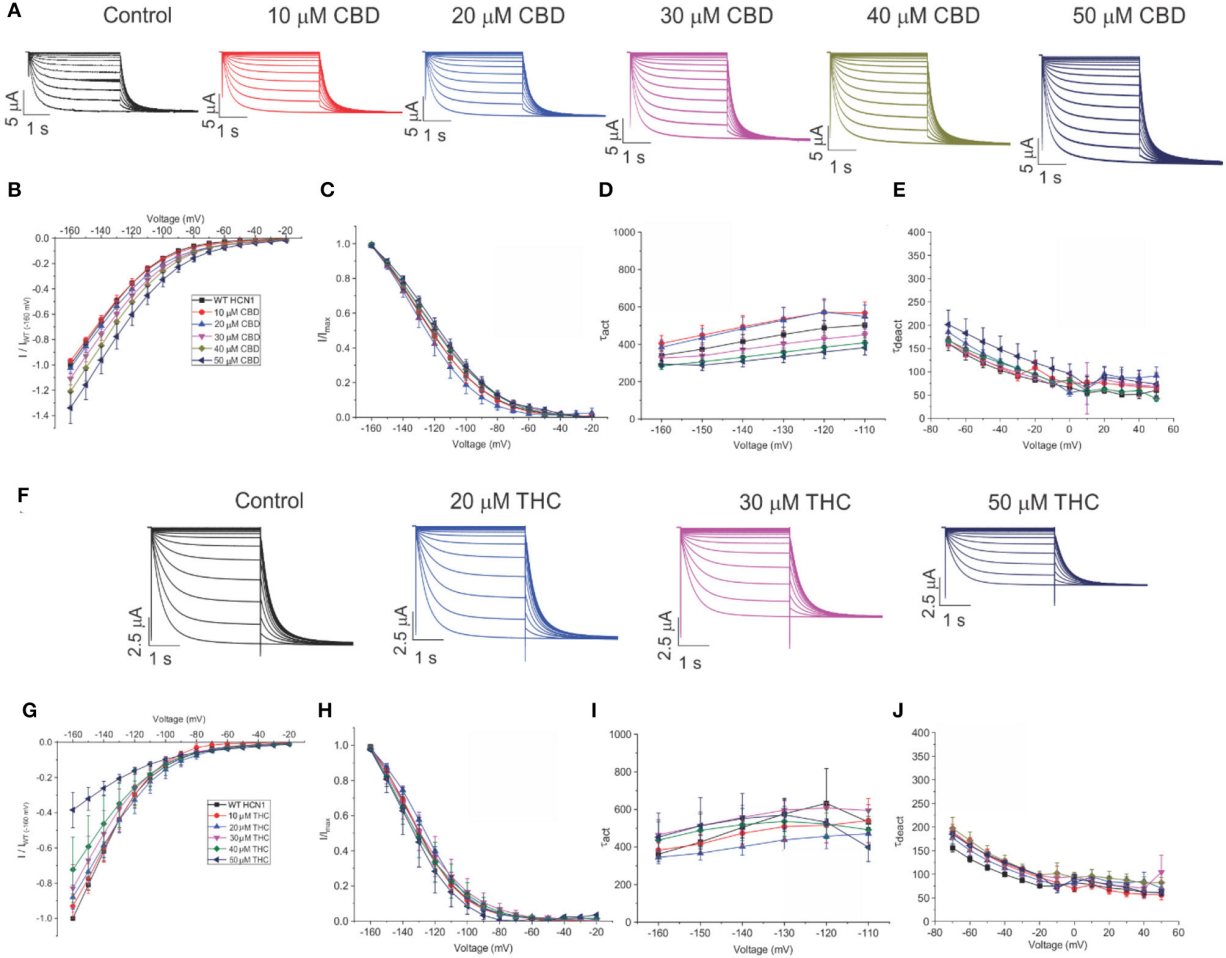
Regulation of HCN1 by cannabidiol (CBD) and Δ^9^-tetrahydrocannabidiol (THC). **(A)** Representative traces from a paired experiment following the addition of increasing concentrations of CBD to oocytes expressing full-length HCN1. **(B)** Current–voltage (I/V) relationship in presence of CBD normalized to maximal current (I_WT(−160mV)_) (5 < *n* <18 per condition; *P* < 0.05 for 30–50 μM). **(C)** Steady state activation in presence of CBD (*P* = 0.81 for V_1/2_). **(D)** Activation time constant (τ) kinetics in presence of CBD (0.21 < *P* < 0.71). **(E)** Deactivation time constant (τ) kinetics in presence of CBD (3 < *n* <7 per condition; 0.09 < *P* <0.65). **(F)** Representative traces from a paired experiment following the addition of increasing concentrations of THC to oocytes expressing full-length HCN1. **(G)** (I/V) relationship in presence of THC normalized to maximal current (I_WT(−160mV)_) (4 < *n* <8 per condition; *P* <0.05 for Gmax (slope between −120 and −160 mV) of 20–50 μM). **(H)** Steady-state activation in presence of THC (*P* = 0.49 for V_1/2_). **(I)** Activation time constant (τ) kinetics in presence of THC (0.21 < *P* < 0.90). **(J)** Deactivation time constant (τ) kinetics in presence of THC (3 < *n* < 8 per condition; 0.11 < *P* < 0.45).

To assess the effects of CBD and THC on HCN1 currents in more detail, we examined the relative I–V relationship, steady-state voltage-dependencies, and gating kinetics. Cannabinoids were added to the bath solution in 10 μM increments only after currents at the previous concentration had stabilized, and for each cell, currents were normalized to the amount of current −160 mV under control (0 μM) conditions. We observed a concentration-dependent increase in HCN1 current with the addition of CBD above 10 μM, with no statistically significant changes in steady-state voltage-dependence and gating kinetics (Figures 3A–E). On the other hand, the addition of THC in the bath induces a concentration-dependent decrease in the current (Figures 3F,G). However, THC also does not affect steady-state voltage-dependence or gating kinetics (Figures 3G,H). Equivalent amounts of methanol used to dissolve CBD or THC have no effect on current, voltage-dependence, or gating kinetics (Supplementary Figure 1).

**Figure 4:**
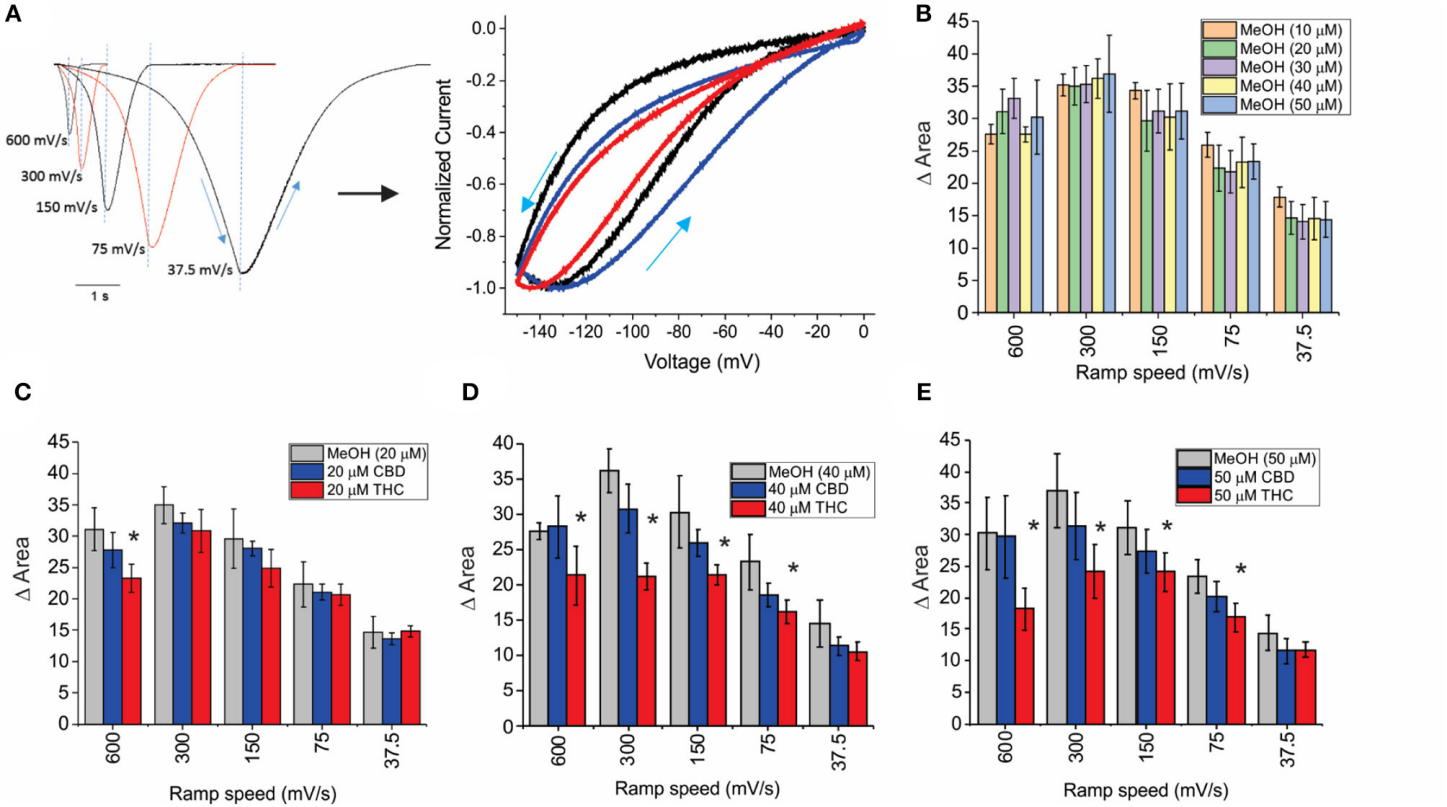
Effects of CBD and THC on HCN1 hysteresis. **(A)** Ramps from 0 to −150 mV and back to 0 mV at speeds of 600, 300, 150, 75, and 37.5 mV/s. The degree of hysteresis was quantified by the area between the forward and backward current traces when plotted vs. the membrane voltage. **(B)** Histogram of HCN1 hysteresis for HCN1 treated with different concentrations of methanol used as vehicles for the cannabinoids at concentrations listed in parentheses. Hysteresis is unaffected by increasing concentrations of methanol. **(C–E)** THC reduces the degree of hysteresis at fast ramp speeds, with a greater range of ramp speeds affected as THC concentration increases. CBD did not affect HCN1 hysteresis (3 < *n* <10 for each condition; *P <0.05).

HCN channels have been shown to undergo a hysteresis or mode-shifting in their voltage-dependence where the voltage sensitivity of gating charge movement depends on the previous state (Mannikko et al., 2005; Elinder et al., 2006; Xiao et al., 2010). Voltage-hysteresis is thought to play an important role in preventing cardiac arrhythmias (Mannikko et al., 2005; Elinder et al., 2006; Xiao et al., 2010), and for short-term, activity-dependent memory (Bruening-Wright and Larsson, 2007). We quantified the degree of hysteresis using ramp protocols of various rates between 600 and 37.5 mV/s and measuring the difference in area between the curves of the forward and reverse direction as performed previously (Furst and D'Avanzo, 2015) (Figure 4A). We observe that HCN1 hysteresis in oocytes decreases with slower ramp speeds (Figure 4A), and that methanol has no concentration-dependent effect on HCN1 hysteresis (Figure 4B). We observe that at all concentrations and ramp speeds, CBD had no significant effect on HCN1 hysteresis (Figures 4C–E). On the other hand, at concentrations above 10 μM, THC decreases the degree of hysteresis at faster ramp speeds (Figures 4C–E). Notably, the impact of THC on HCN1 hysteresis also appears to be concentration-dependent with a greater range of ramp speeds affected as THC concentrations increase.

**Figure 5:**
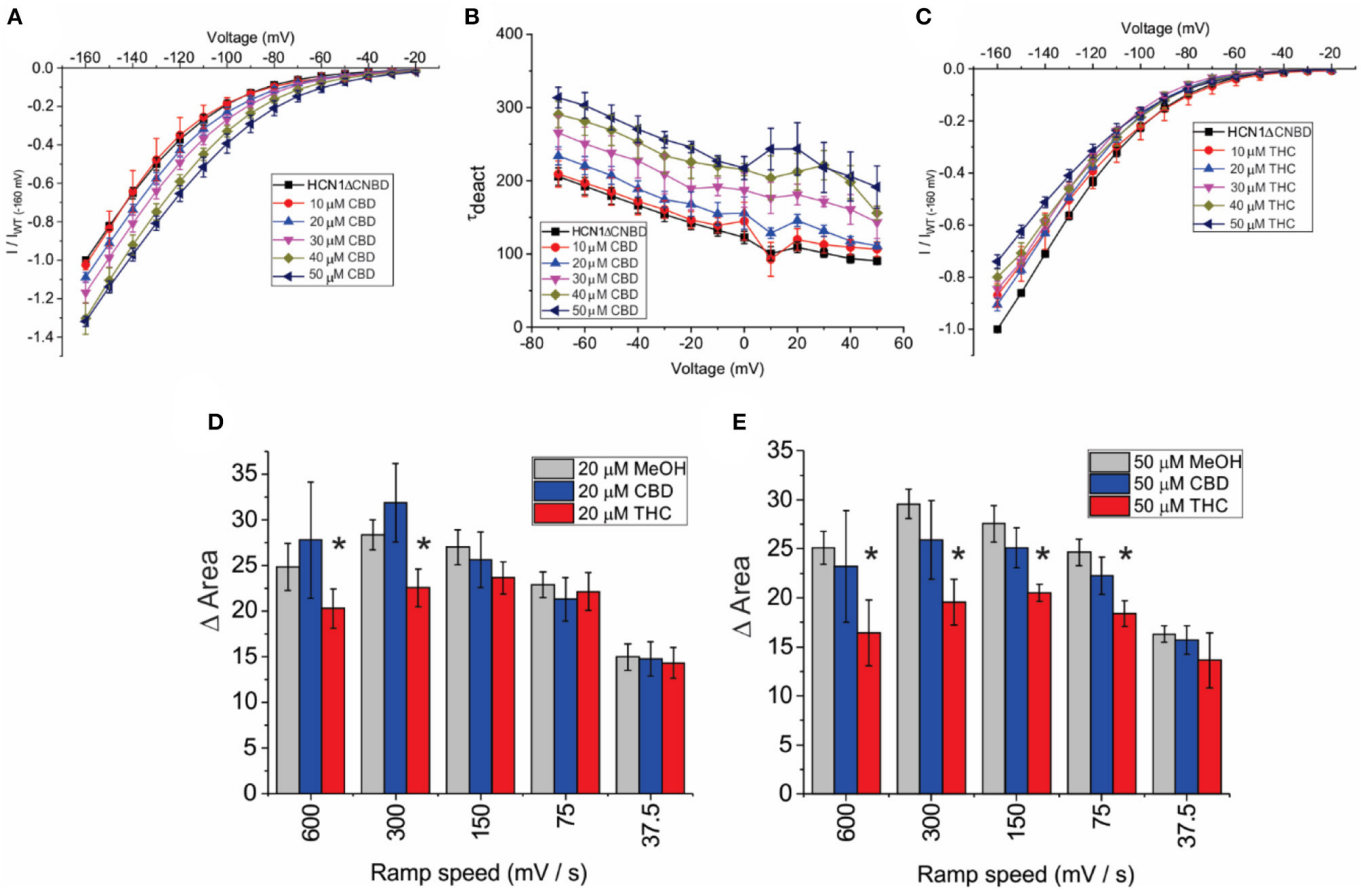
Effects of CBD and THC on HCN1 do not require the CNBD. **(A)** Current-voltage (I/V) relationship of HCN1ΔCNBD in the presence of increasing concentrations of CBD normalized to maximal current (I_WT(−160mV)_) (4 < *n* < 13 per condition; *P* < 0.05 for 20–50 μM). **(B)** Deactivation time constant (τ) kinetics of HCN1ΔCNBD following treatment with CBD (4 < *n* <10 per condition; *P* < 0.05 for 20–50 μM). **(C)** Current-voltage (I/V) relationship of HCN1ΔCNBD in the presence of increasing concentrations of THC normalized to maximal current (I_WT(−160*mV*)_) (4 < *n* <12 per condition; *P* < 0.05 for 10–50 μM). **(D,E)** THC reduces the degree of HCN1ΔCNBD hysteresis at fast ramp speeds, with a greater range of ramp speeds affected as THC concentration increases. CBD did not affect HCN1ΔCNBD hysteresis (3 < *n* <10 for each condition; *P < 0.05).

To address the mechanism(s) by which CBD and THC modulate HCN1 channels, we first examined the role of the CNBD, by performing similar experiments on an mHCN1 construct lacking the cyclic-nucleotide binding domain (HCN1ΔCNBD) (Santoro et al., 2011). Similar to full-length channels, methanol has no effect on HCN1ΔCNBD properties (Supplementary Figures 1E–H). The application of CBD to HCN1ΔCNBD channels increases the current in a concentration-dependent manner, with a 35% maximal increase in current above 40 μM (Figure 5A). Steady-state voltage dependence and activation kinetics are unaffected by treatment with CBD (Supplementary Figures 2B,C). Intriguingly, the lack of CNBD uncovered an effect of CBD on slowing the deactivation kinetics of HCN1 (Figure 5B). On the other hand, HCN1ΔCNBD current decreases following treatment with THC, although the effect on slope conductance (Figure 5C) is less than what was observed for full-length HCN1 (Figure 3G). Again, similar to full-length channels, THC does not affect HCN1 steady-state voltage-dependence and gating kinetics (Supplementary Figures 2F–H). Hysteresis in HCN1ΔCNBD channels is also unaffected by methanol and CBD, however, similarly to what we observed for full-length channels, HCN1ΔCNBD hysteresis also decreases with elevated levels of THC at faster ramp speeds (Figures 5D,E). These results suggest that the primary effects of CBD and THC on HCN1 function do not directly involve the CNBD, though this domain may fine tune their modulation.

**Figure 6:**
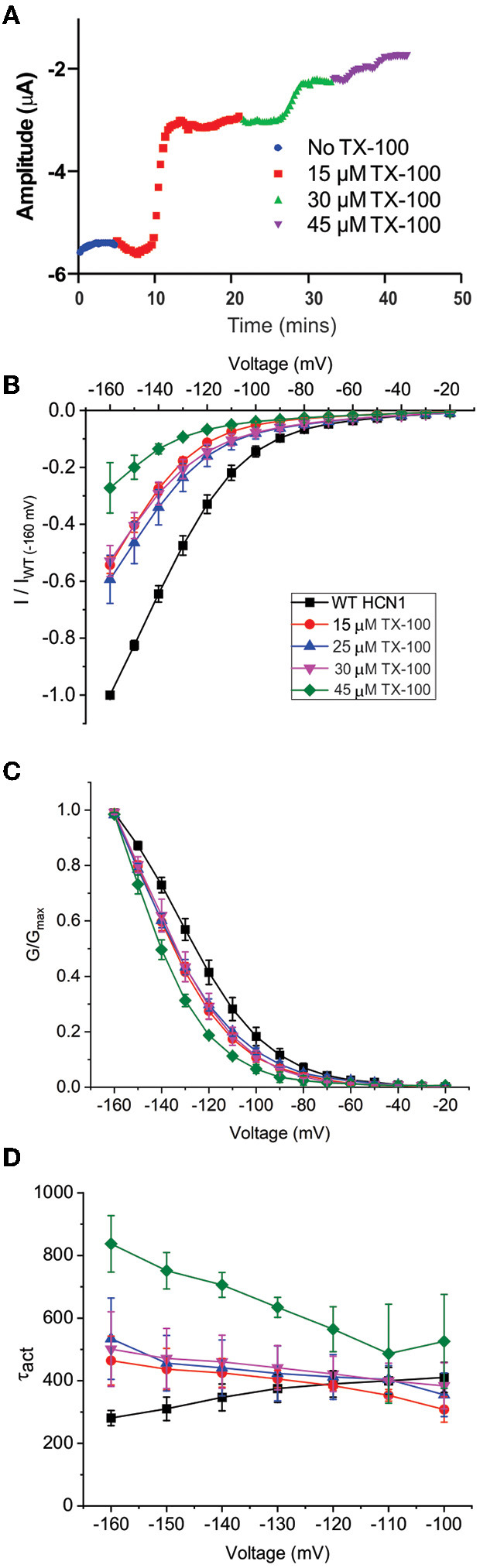
Changes in membrane fluidity do not account for cannabinoid regulation of HCN1. Membrane fluidity was altered by the addition of Triton X-100 (TX-100) to the bath solution. **(A)** Representative time course of the steady-state current following the application of TX-100 at different concentrations, indicating that increasing membrane fluidity decreases HCN1 channel activity. However, this decrease in HCN1 current occurs more rapidly (within 1–2 min) than the decline observed with THC (which occurs over the course of 10–30 min). **(B)** Current-voltage relationship of HCN1 upon the addition of 0, 15, 25, 30, and 45 μM TX-100 normalized to maximal current (I_WT(−160mV)_). **(C)** TX-100 induces a −10 to −15 mV hyperpolarizing shift in the steady-state voltage-dependence of HCN1, which does not occur when THC or CBD is applied. **(D)** HCN1 activation kinetics are uniquely affected by TX-100, with membrane fluidity having a greater impact on slowing channel activation with more hyperpolarization of the membrane potential (3 < *n* <9 per condition; *P* < 0.05).

Our results suggest that the CBR-independent regulation of HCN1 channels by CBD and THC does not involve the CNBD and thus likely involves the transmembrane regions. The functions of numerous membrane proteins are regulated by the physiochemical properties of the membrane bilayer, including membrane fluidity (Caires et al., 2017; Yoshida et al., 2019; Ghovanloo et al., 2021). CBD and THC have both been shown to alter membrane fluidity. THC increases membrane fluidity in the hydrophobic core of brain membranes (Hillard et al., 1985; Beiersdorf et al., 2020), while CBD decreases membrane fluidity (increases membrane stiffness) (Ghovanloo et al., 2021). To assess whether the mechanism of cannabinoid regulation of HCN1 involves changes to membrane fluidity, we performed experiments using Triton X-100, a non-ionic surfactant that has been shown to increase membrane fluidity (Ingolfsson et al., 2010). Similar to THC, TX-100 rapidly decreases HCN1 current at −130 mV using a repetitive pulse protocol (Figure 6A). However, while the effects of cannabinoid treatment on HCNs were slow (15–40 min), the effects of TX-100 treatment occurred much more rapidly (<5 min). TX-100 also decreases HCN1 current using step protocols (Figure 6B), however, the effects on steady-state activation and gating kinetics are significantly different compared with THC (e.g., *P* <0.05 for V_1/2_ of 30 μM THC vs. TX-100 by two-sample unpaired *t*-test). We see a leftward shift from −10 to −15 mV in the steady-state, meaning increasing membrane fluidity makes it more difficult for HCN1 channels to open (Figure 6C and Supplementary Table 3). In addition, we see a change in the slope of the activation time constants, indicating that the channel opens slower at more hyperpolarized voltages (Figure 6D). This change in slope of the activation time constants was not observed in cannabinoid treated cells. Thus, since the effects of TX-100 do not resemble the modulation seen by either CBD or THC, it appears that the mechanism of HCN1 regulation by either of these cannabinoids cannot be completely described by the effects of altered membrane fluidity.

## Discussion

Phytocannabinoids CBD and THC have been shown to interact with numerous ion channels independently of CBRs (Okada et al., 2005; Hejazi et al., 2006; Ross et al., 2008; Ahrens et al., 2009; De Petrocellis et al., 2011, 2012; Iannotti et al., 2014; Ghovanloo et al., 2018). Here, we demonstrate that HCN1 currents are enhanced by CBD and inhibited by THC (Figures 2, 3). Using standard step protocols, the effects of both CBD and THC were limited to changes in current density with no observed changes in steady-state activation or gating kinetics. This is similar to the effects that modulating membrane cholesterol content has on HCN1 current (Furst and D'Avanzo, 2015). Some of the effects of THC and CBD on voltage-dependence may be obscured by the non-saturating voltage-dependence curve we report, as a result of our use of a high K^+^ bath solution. However, if the basis of the increase in HCN1 current observed following treatment with CBD was caused a shift in voltage-dependence, we would expect the V_1/2_ to be more depolarized and thus saturation to be more apparent. From our traces and the data, we see this is not the case. On the other hand, it is possible that a further hyperpolarized shift in V_1/2_ may cause the effect of decreased HCN1 currents by THC. However, this is also unlikely since we were able to detect such a hyperpolarizing shift with TX-100 but not THC. Thus, it is more likely that the number of channels at the membrane, or the unitary conductance, are affected by treatment by these cannabinoids.

We also observed that THC alters the non-equilibrium gating properties of the HCN1, since they undergo less hysteresis following treatment with that cannabinoid, particularly with faster ramps (Figure 4). This means THC makes HCN1 channels less responsive to the rate of membrane potential changes (i.e., firing rate). The CNBD domain does not appear to be critical for cannabinoid regulation of HCN function, since these changes are also observed in recordings of HCN1ΔCNBD channels (Figure 5). This also indicates that CBD and THC do not alter HCN1 channels through changes in intracellular cyclic-nucleotide signaling. However, the lack of the CNBD did unmask the effects of CBD on the HCN deactivation kinetics (Figure 5B), indicating that CBD in the membrane slows channel closure. Since cannabinoids are lipids that are embedded in the lipid bilayer, they can alter channel function by altering membrane properties and/or directly binding the channel. Both mechanisms appear to be important for the regulation of Nav's by CBD (Ghovanloo et al., 2018, 2021; Sait et al., 2020) while TRPV2 directly binds CBD in the transmembrane helices (Pumroy et al., 2019). Changes to membrane fluidity were induced by the treatment of cells by TX-100, a non-ionic surfactant that has been shown to increase membrane fluidity (Ingolfsson et al., 2010). TX-100 decreases HCN1 function, indicating that membrane fluidity is an important modulator of this channel. However, the rate of action and the changes in voltage-dependent activation and gating kinetics did not reflect the effects of CBD or THC on HCN1 that we observe. While it is possible that TX-100 has greater effects on membrane fluidity than THC at the concentrations used, even at low (15 μM) concentrations, the effects of TX-100 on HCN1 currents are faster than THC, a −10 mV change in V_1/2_ is observed, and the slopes of the activation kinetics are in the opposite direction. THC does not induce these effects even at the highest concentrations used, and thus their effects appear qualitatively different. Therefore, it appears that the primary mechanism of action on HCN1 channels is not through altered membrane fluidity, though it may still be a secondary contributor to the effects observed. However, at this time, we cannot conclude that these phytocannabinoids directly bind HCN channels without further experimentation. Other mechanisms, such as changes in bilayer thickness/hydrophobic matching, lipid organization, or other membrane properties may contribute to the modulation by cannabinoids. Since the macroscopic current is a function of the unitary conductance, the number of channels, and the open probability, we did not observe changes in steady-state voltage dependence (i.e., open probability), meaning THC and CBD may alter the single-channel properties of these channels. Alternatively, given the slow time courses involved in the observed effects, it is possible that these cannabinoids alter protein turnover at the membrane, altering the number of channels at the membrane with time.

The concentrations of CBD and THC necessary to affect HCN1 channels (in the mid-micromolar range) may be considered high for these molecules to have therapeutic actions through these channels. However, additional considerations may prove such concerns to be unwarranted. At first, various other ion channels have been shown to be modulated by CBD and THC within the concentration ranges used in this study. Direct activation of chloride ion channels (α_1_ and α_2_β glycine receptors) by CBD has been reported with an EC_50_ of 132.4 and 144.3 μM (Ahrens et al., 2009). Sodium channel, NavMs, and α_7_ -nicotinic acethylcholine (α_7_ nACh) are inhibited by CBD with an IC_50_ of 17.8 (Sait et al., 2020) and 11.3 μM (Mahgoub et al., 2013), respectively. Kv1.2 channels are inhibited by THC with an IC_50_ of 2.4 μM (Poling et al., 1996), while human ether-à-go-go (hERG) channels are inhibited with an IC_50_ of 10.3 μM (Orvos et al., 2020). In addition, single-dose administration of 10 mg CBD and THC generate serum levels of 3.0 ± 3.1μg/L (= 9.1–19.4 μM) (Guy and Flint, 2004; Guy and Robson, 2004). Anticonvulsant effects of THC and CBD have an ED_50_ ~120 mg/kg (Devinsky et al., 2014). Depending on the mode of administration, 120 mg/kg of CBD leads to concentrations of 7 μM in serum and 1.3 μg/g in the brain; increased with IP administration to 45 μM in serum and 6.9 μg/g in the brain (Deiana et al., 2012). Because of their high lipophilicity (K_octanol−water_ ~ 6–7), there is a preferential distribution to fat with rapid distribution in the brain, adipose tissue, and other organs (Ohlsson et al., 1984) with only 10% of administered CBD bound to circulating red blood cells (Williamson, 2004). Chronic administration may lead to further accumulation. It is important to also note the relative differences in IC_50_ and EC_50_ values between mammalian cell lines such as HEK or CHO cells and *Xenopus* oocytes observed for some drugs, including cannabinoids. Previous studies reveal IC_50_ and EC_50_ values which are significantly higher in *Xenopus* oocytes when compared with mammalian cells. Potassium channel (Kv1.1) blocker, aminopyridine (4-AP), was shown to inhibit channels expressed in mammalian Sol-8 cells with an IC_50_ value of 88 ± 5 μM (Castle et al., 1994). This value was more than 10 times higher in oocytes with IC_50_ values closer to 1 mM. Another study monitoring the efficacy of various blocking agents on hERG potassium channels showed 5–100 times higher IC_50_ values in *Xenopus* oocytes when compared with the mammalian HEK293 and CHO cells (Lacerda et al., 2001). K2P10.1 channels are blocked by carvedilol with an IC_50_ of 24 μM in oocytes and 7.6 μM in HEK cells (Kisselbach et al., 2014). In our experiments, TRPV1 channels in oocytes required more than 10 μM CBD before activation could be observed (Supplementary Figure 3) whereas an EC_50_ of 1.0 ± 0.1 μM was reported in HEK-293 cells (De Petrocellis et al., 2011). These differences between cell types might be attributed to differences in basal properties of the cells (e.g., differences in levels of cAMP, or phosphorylation) or to differences in the membrane composition which may modify the equilibrium of cannabinoid insertion into the bilayers. For example, *Xenopus* oocytes membranes possess higher levels of sphingomyelin (~25% of lipids) (Stith et al., 2000; Hermansson et al., 2005; Hill et al., 2005; Pike et al., 2005) in their external leaflet than mammalian cells (4–18%) (Post et al., 1995; Hamplova et al., 2004; Hermansson et al., 2005; Pike et al., 2005). Moreover, some drugs have been shown to also bind the follicular layer of oocytes, with as much as a 90% reduction in membrane availability and increases of the IC_50_ values up to 30-fold (Madeja et al., 1997). Therefore, the general effect of the ligand still holds true, and variations between cell types are generally limited to 1 order of magnitude. Taking this into account, it is likely the effects of CBD and THC we observe for HCN1 channels in oocytes are not outside the therapeutic range, especially since they are comparable to those observed for other channels and receptors, and that the IC_50_ and EC_50_ values are likely to be the same, if not even lower, in the mammalian cells.

Cannabinoids are already being examined for the therapeutic potential of various neurological disorders, including neuropathic pain and epileptic seizures (Jones et al., 2010). Similarly, HCN channels are promising targets for neuropathic pain and epilepsy (Tibbs et al., 2013; Dini et al., 2018; Balducci et al., 2021). Activation of HCN1 current may contribute to the overall reduction of neuronal hyperactivity in epilepsy with CBD treatment (Iannotti et al., 2014; Maroon and Bost, 2018), similar to the reduced neuronal firing observed in CA1 pyramidal neurons resulting from lamotrigine stimulated I_h_ (Peng et al., 2010). There is also growing evidence that CBD exerts promising analgesic effects in different models of inflammatory and chronic pain including nerve injury, chemotherapy-induced peripheral neuropathy, and diabetes (Costa et al., 2007; Xiong et al., 2012; Casey et al., 2017; Finn et al., 2021). THC also significantly attenuates pain-related behaviors in nerve injury models (Soliman et al., 2021). In addition to animal studies, clinical studies have demonstrated that a combination of THC and CBD can be an effective therapeutic option for patients with neuropathic and other types of chronic pain (Nurmikko et al., 2007; Turcotte et al., 2010; Lynch and Campbell, 2011). Since HCN1 expression and I_h_ in HCN1/2-rich sensory neurons increase following their injury (Momin et al., 2008) and antineoplastics treatment (Descoeur et al., 2011), or HCN pore blockers (ZD7288 or ivabradine) reverse spontaneous discharges in injured nerve fibers and are anti-hyperalgesic for late-phase inflammatory pain, nerve injury-induced mechanical allodynia, as well as chemotherapy-induced mechanical and thermal hyperalgesia (Tibbs et al., 2016), we anticipate that CBD and THC effects on HCN1 currents may contribute to their role in reducing pain though through different mechanisms. Inhibition of HCN1 current by THC would be expected to reduce excitability in those neurons, as has been demonstrated for other HCN blockers. On the other hand, stimulation of HCN1 by CBD may reduce pain by depleting neurotransmitter release in sensory neurons similar to capsaicin stimulation of TRPV channels (Willis, 1997; Yan et al., 2006) or by stimulating inhibition by GABAergic interneurons. On the other hand, activation of HCN channels by CBD may not be directly involved in analgesia, but rather may help limit the decrease in excitability induced by action on other channels, including Nav's, whose conductance is inhibited up to 90% (Ghovanloo et al., 2018, 2021).

## Data Availability Statement

The raw data supporting the conclusions of this article will be made available by the authors, without undue reservation.

## Author Contributions

Experiments were performed and analyzed by SM, MM-Y, AM, RE, and ND'A. The manuscript was prepared by SM and ND'A. All authors contributed to the article and approved the submitted version.

## Funding

This work was supported by a Discovery Grant (RGPIN-2019-00373) from the National Science and Engineering Research Council (NSERC) and a Project Grant from the Canadian Institutes of Health Research (CIHR) (FRN 173388) awarded to ND'A.

## Conflict of Interest

The authors declare that the research was conducted in the absence of any commercial or financial relationships that could be construed as a potential conflict of interest.

## Publisher's Note

All claims expressed in this article are solely those of the authors and do not necessarily represent those of their affiliated organizations, or those of the publisher, the editors and the reviewers. Any product that may be evaluated in this article, or claim that may be made by its manufacturer, is not guaranteed or endorsed by the publisher.

## References

[B1] Abdulla F. A. Smith P. A. (2001). Axotomy- and autotomy-induced changes in Ca2+ and K+ channel currents of rat dorsal root ganglion neurons. J. Neurophysiol. 85, 644–658. 10.1152/jn.2001.85.2.644 11160500

[B2] Ahrens J. Demir R. Leuwer M. de la Roche J. Krampfl K. Foadi N.. (2009). The nonpsychotropic cannabinoid cannabidiol modulates and directly activates alpha-1 and alpha-1-beta glycine receptor function. Pharmacology 83, 217–222. 10.1159/000201556 19204413

[B3] Arnsten A. F.. (2011). Prefrontal cortical network connections: key site of vulnerability in stress and schizophrenia. Int. J. Dev. Neurosci. 29, 215–223. 10.1016/j.ijdevneu.2011.02.006 21345366 PMC3115784

[B4] Balducci V. Credi C. Sacconi L. Romanelli M. N. Sartiani L. Cerbai E. (2021). The HCN channel as a pharmacological target: why, where, and how to block it. Prog. Biophys. Mol. Biol. 166, 173–181. 10.1016/j.pbiomolbio.2021.07.010 34303730

[B5] Beiersdorf J. Hevesi Z. Calvigioni D. Pyszkowski J. Romanov R. Szodorai E.. (2020). Adverse effects of Delta9-tetrahydrocannabinol on neuronal bioenergetics during postnatal development. JCI Insight 5:e135418. 10.1172/jci.insight.135418 33141759 PMC7714410

[B6] Bruening-Wright A. Larsson H. P. (2007). Slow conformational changes of the voltage sensor during the mode shift in hyperpolarization-activated cyclic-nucleotide-gated channels. J. Neurosci. 27, 270–278. 10.1523/JNEUROSCI.3801-06.2007 17215386 PMC6672073

[B7] Caires R. Sierra-Valdez F. J. Millet J. R. M. Herwig J. D. Roan E. Vasquez V.. (2017). Omega-3 fatty acids modulate TRPV4 function through plasma membrane remodeling. Cell Rep. 21, 246–258. 10.1016/j.celrep.2017.09.029 28978477 PMC8611619

[B8] Cardenas C. G. Mar L. P. Vysokanov A. V. Arnold P. B. Cardenas L. M. Surmeier D. J.. (1999). Serotonergic modulation of hyperpolarization-activated current in acutely isolated rat dorsal root ganglion neurons. J. Physiol. 518, 507–523. 10.1111/j.1469-7793.1999.0507p.x 10381596 PMC2269436

[B9] Casey S. L. Atwal N. Vaughan C. W. (2017). Cannabis constituent synergy in a mouse neuropathic pain model. Pain 158, 2452–2460. 10.1097/j.pain.0000000000001051 28885457

[B10] Castle N. A. Fadous S. Logothetis D. E. Wang G. K. (1994). Aminopyridine block of Kv1.1 potassium channels expressed in mammalian cells and Xenopus oocytes. Mol. Pharmacol. 45:1242. 8022416

[B11] Chaplan S. R. Guo H. Q. Lee D. H. Luo L. Liu C. Kuei C.. (2003). Neuronal hyperpolarization-activated pacemaker channels drive neuropathic pain. J. Neurosci. 23, 1169–1178. 10.1523/JNEUROSCI.23-04-01169.2003 12598605 PMC6742242

[B12] Costa B. Trovato A. E. Comelli F. Giagnoni G. Colleoni M. (2007). The non-psychoactive cannabis constituent cannabidiol is an orally effective therapeutic agent in rat chronic inflammatory and neuropathic pain. Eur. J. Pharmacol. 556, 75–83. 10.1016/j.ejphar.2006.11.006 17157290

[B13] Cunha J. M. Carlini E. A. Pereira A. E. Ramos O. L. Pimentel C. Gagliardi R.. (1980). Chronic administration of cannabidiol to healthy volunteers and epileptic patients. Pharmacology 21, 175–185. 10.1159/000137430 7413719

[B14] De Petrocellis L. Ligresti A. Moriello A. S. Allara M. Bisogno T. Petrosino S.. (2011). Effects of cannabinoids and cannabinoid-enriched Cannabis extracts on TRP channels and endocannabinoid metabolic enzymes. Br. J. Pharmacol. 163, 1479–1494. 10.1111/j.1476-5381.2010.01166.x 21175579 PMC3165957

[B15] De Petrocellis L. Orlando P. Moriello A. S. Aviello G. Stott C. Izzo A. A.. (2012). Cannabinoid actions at TRPV channels: effects on TRPV3 and TRPV4 and their potential relevance to gastrointestinal inflammation. Acta Physiol. 204, 255–266. 10.1111/j.1748-1716.2011.02338.x 21726418

[B16] Deiana S. Watanabe A. Yamasaki Y. Amada N. Arthur M. Fleming S.. (2012). Plasma and brain pharmacokinetic profile of cannabidiol (CBD), cannabidivarine (CBDV), Δ?-tetrahydrocannabivarin (THCV) and cannabigerol (CBG) in rats and mice following oral and intraperitoneal administration and CBD action on obsessive-compulsive behaviour. Psychopharmacology 219, 859–873. 10.1007/s00213-011-2415-0 21796370

[B17] Descoeur J. Pereira V. Pizzoccaro A. Francois A. Ling B. Maffre V.. (2011). Oxaliplatin-induced cold hypersensitivity is due to remodelling of ion channel expression in nociceptors. EMBO Mol. Med. 3, 266–278. 10.1002/emmm.201100134 21438154 PMC3377073

[B18] Devinsky O. Cilio M. R. Cross H. Fernandez-Ruiz J. French J. Hill C.. (2014). Cannabidiol: pharmacology and potential therapeutic role in epilepsy and other neuropsychiatric disorders. Epilepsia 55, 791–802. 10.1111/epi.12631 24854329 PMC4707667

[B19] Dibbens L. M. Reid C. A. Hodgson B. Thomas E. A. Phillips A. M. Gazina E.. (2010). Augmented currents of an HCN2 variant in patients with febrile seizure syndromes. Ann. Neurol. 67, 542–546. 10.1002/ana.21909 20437590 PMC3383007

[B20] DiFrancesco J. C. Barbuti A. Milanesi R. Coco S. Bucchi A. Bottelli G.. (2011). Recessive loss-of-function mutation in the pacemaker HCN2 channel causing increased neuronal excitability in a patient with idiopathic generalized epilepsy. J. Neurosci. 31, 17327–17337. 10.1523/JNEUROSCI.3727-11.2011 22131395 PMC6623833

[B21] Dini L. Del Lungo M. Resta F. Melchiorre M. Spinelli V. Di Cesare Mannelli L.. (2018). Selective Blockade of HCN1/HCN2 channels as a potential pharmacological strategy against pain. Front. Pharmacol. 9:1252. 10.3389/fphar.2018.01252 30467478 PMC6237106

[B22] Elinder F. Mannikko R. Pandey S. Larsson H. P. (2006). Mode shifts in the voltage gating of the mouse and human HCN2 and HCN4 channels. J Physiol. 575, 417–431. 10.1113/jphysiol.2006.110437 16777944 PMC1819464

[B23] Emery E. C. Young G. T. Berrocoso E. M. Chen L. McNaughton P. A. (2011). HCN2 ion channels play a central role in inflammatory and neuropathic pain. Science 333, 1462–1466. 10.1126/science.1206243 21903816

[B24] Finn D. P. Haroutounian S. Hohmann A. G. Krane E. Soliman N. Rice A. S. C. (2021). Cannabinoids, the endocannabinoid system, and pain: a review of preclinical studies. Pain 162, S5–S25. 10.1097/j.pain.0000000000002268 33729211 PMC8819673

[B25] Furst O. D'Avanzo N. (2015). Isoform dependent regulation of human HCN channels by cholesterol. Sci. Rep. 5:14270. 10.1038/srep14270 26404789 PMC4585891

[B26] Gamo N. J. Lur G. Higley M. J. Wang M. Paspalas C. D. Vijayraghavan S.. (2015). Stress impairs prefrontal cortical function via D1 dopamine receptor interactions with hyperpolarization-activated cyclic nucleotide-gated channels. Biol. Psychiatry 78, 860–870. 10.1016/j.biopsych.2015.01.009 25731884 PMC4524795

[B27] Ghovanloo M. R. Choudhury K. Bandaru T. S. Fouda M. A. Rayani K. Rusinova R.. (2021). Cannabidiol inhibits the skeletal muscle Nav1.4 by blocking its pore and by altering membrane elasticity. J. Gen. Physiol. 153:e202012701. 10.1085/jgp.202012701 33836525 PMC8042605

[B28] Ghovanloo M. R. Shuart N. G. Mezeyova J. Dean R. A. Ruben P. C. Goodchild S. J. (2018). Inhibitory effects of cannabidiol on voltage-dependent sodium currents. J. Biol. Chem. 293, 16546–16558. 10.1074/jbc.RA118.004929 30219789 PMC6204917

[B29] Guy G. W. Flint M. E. (2004). A single centre, placebo-controlled, four period, crossover, tolerability study assessing, pharmacodynamic effects, pharmacokinetic characteristics and cognitive profiles of a single dose of three formulations of Cannabis Based Medicine Extracts (CBMEs) (GWPD9901), plus a two period tolerability study comparing pharmacodynamic effects and pharmacokinetic characteristics of a single dose of a cannabis based medicine extract given via two administration routes (GWPD9901 EXT). J. Cannabis Ther. 3, 35–77. 10.1300/J175v03n03_03

[B30] Guy G. W. Robson P. J. (2004). A phase I, open label, four-way crossover study to compare the pharmacokinetic profiles of a single dose of 20 mg of a Cannabis Based Medicine Extract (CBME) Administered on 3 different areas of the buccal mucosa and to investigate the pharmacokinetics of CBME per oral in healthy male and female volunteers (GWPK0112). J. Cannabis Ther. 3, 79–120. 10.1300/J175v03n04_01

[B31] Hamplova B. Pelouch V. Novakova O. Skovranek J. Hucin B. Novak F. (2004). Phospholipid composition of myocardium in children with normoxemic and hypoxemic congenital heart diseases. Physiol. Res. 53, 557–560. 15479135

[B32] Hejazi N. Zhou C. Oz M. Sun H. Ye J. H. Zhang L. (2006). Delta9-tetrahydrocannabinol and endogenous cannabinoid anandamide directly potentiate the function of glycine receptors. Mol. Pharmacol. 69, 991–997. 10.1124/mol.105.019174 16332990

[B33] Hermansson M. Uphoff A. Kakela R. Somerharju P. (2005). Automated quantitative analysis of complex lipidomes by liquid chromatography/mass spectrometry. Anal. Chem. 77, 2166–2175. 10.1021/ac048489s 15801751

[B34] Hill A. J. Williams C. M. Whalley B. J. Stephens G. J. (2012). Phytocannabinoids as novel therapeutic agents in CNS disorders. Pharmacol. Ther. 133, 79–97. 10.1016/j.pharmthera.2011.09.002 21924288

[B35] Hill W. G. Southern N. M. MacIver B. Potter E. Apodaca G. Smith C. P.. (2005). Isolation and characterization of the Xenopus oocyte plasma membrane: a new method for studying activity of water and solute transporters. Am. J. Physiol. Renal Physiol. 289, F217–F224. 10.1152/ajprenal.00022.2005 15741609

[B36] Hillard C. J. Harris R. A. Bloom A. S. (1985). Effects of the cannabinoids on physical properties of brain membranes and phospholipid vesicles: fluorescence studies. J. Pharmacol. Exp. Ther. 232, 579–588. 2983062

[B37] Huang Z. Walker M. C. Shah M. M. (2009). Loss of dendritic HCN1 subunits enhances cortical excitability and epileptogenesis. J. Neurosci. 29, 10979–10988. 10.1523/JNEUROSCI.1531-09.2009 19726656 PMC2744118

[B38] Iannotti F. A. Hill C. L. Leo A. Alhusaini A. Soubrane C. Mazzarella E.. (2014). Nonpsychotropic plant cannabinoids, cannabidivarin (CBDV) and cannabidiol (CBD), activate and desensitize transient receptor potential vanilloid 1 (TRPV1) channels in vitro: potential for the treatment of neuronal hyperexcitability. ACS Chem. Neurosci. 5, 1131–1141. 10.1021/cn5000524 25029033

[B39] Ingolfsson H. I. Sanford R. L. Kapoor R. Andersen O. S. (2010). Gramicidin-based fluorescence assay; for determining small molecules potential for modifying lipid bilayer properties. J. Vis. Exp. 44:e2131. 10.3791/2131 20972414 PMC3185632

[B40] Jones N. A. Hill A. J. Smith I. Bevan S. A. Williams C. M. Whalley B. J.. (2010). Cannabidiol displays antiepileptiform and antiseizure properties *in vitro* and *in vivo*. J. Pharmacol. Exp. Ther. 332, 569–577. 10.1124/jpet.109.159145 19906779 PMC2819831

[B41] Karimi K. Fortriede J. D. Lotay V. S. Burns K. A. Wang D. Z. Fisher M. E.. (2018). Xenbase: a genomic, epigenomic and transcriptomic model organism database. Nucleic Acids Res. 46, D861–D868. 10.1093/nar/gkx936 29059324 PMC5753396

[B42] Kisselbach J. Seyler C. Schweizer P. A. Gerstberger R. Becker R. Katus H. A.. (2014). Modulation of K2P 2.1 and K2P 10.1 K(+) channel sensitivity to carvedilol by alternative mRNA translation initiation. Br. J. Pharmacol. 171, 5182–5194. 10.1111/bph.12596 25168769 PMC4294033

[B43] Lacerda A. E. Kramer J. Shen K. Z. Thomas D. Brown A. M. (2001). Comparison of block among cloned cardiac potassium channels by non-antiarrhythmic drugs. Eur. Heart J. Suppl. 3, K23–K30. 10.1016/S1520-765X(01)90003-3

[B44] Ludwig A. Budde T. Stieber J. Moosmang S. Wahl C. Holthoff K.. (2003). Absence epilepsy and sinus dysrhythmia in mice lacking the pacemaker channel HCN2. EMBO J. 22, 216–224. 10.1093/emboj/cdg032 12514127 PMC140107

[B45] Ludwig A. Zong X. Jeglitsch M. Hofmann F. Biel M. (1998). A family of hyperpolarization-activated mammalian cation channels. Nature 393, 587–591. 10.1038/31255 9634236

[B46] Lynch M. E. Campbell F. (2011). Cannabinoids for treatment of chronic non-cancer pain; a systematic review of randomized trials. Br. J. Clin. Pharmacol. 72, 735–744. 10.1111/j.1365-2125.2011.03970.x 21426373 PMC3243008

[B47] Madeja M. Musshoff U. Speckmann E. J. (1997). Follicular tissues reduce drug effects on ion channels in oocytes of Xenopus laevis. Eur. J. Neurosci. 9, 599–604. 10.1111/j.1460-9568.1997.tb01636.x 9104601

[B48] Mahgoub M. Keun-Hang S. Y. Sydorenko V. Ashoor A. Kabbani N. Al Kury L.. (2013). Effects of cannabidiol on the function of α7-nicotinic acetylcholine receptors. Eur. J. Pharmacol. 720, 310–319. 10.1016/j.ejphar.2013.10.011 24140434

[B49] Mannikko R. Pandey S. Larsson H. P. Elinder F. (2005). Hysteresis in the voltage dependence of HCN channels: conversion between two modes affects pacemaker properties. J. Gen. Physiol. 125, 305–326. 10.1085/jgp.200409130 15710913 PMC2234019

[B50] Maroon J. Bost J. (2018). Review of the neurological benefits of phytocannabinoids. Surg. Neurol. Int. 9:91. 10.4103/sni.sni_45_18 29770251 PMC5938896

[B51] Maroso M. Szabo G. G. Kim H. K. Alexander A. Bui A. D. Lee S. H.. (2016). Cannabinoid control of learning and memory through HCN Channels. Neuron 89, 1059–1073. 10.1016/j.neuron.2016.01.023 26898775 PMC4777634

[B52] Masuda N. Hayashi Y. Matsuyoshi H. Chancellor M. B. de Groat W. C. Yoshimura N. (2006). Characterization of hyperpolarization-activated current (Ih) in dorsal root ganglion neurons innervating rat urinary bladder. Brain Res. 1096, 40–52. 10.1016/j.brainres.2006.04.085 16765328

[B53] Momin A. Cadiou H. Mason A. McNaughton P. A. (2008). Role of the hyperpolarization-activated current Ih in somatosensory neurons. J Physiol. 586, 5911–5929. 10.1113/jphysiol.2008.163154 18936078 PMC2655434

[B54] Moosmang S. Stieber J. Zong X. Biel M. Hofmann F. Ludwig A. (2001). Cellular expression and functional characterization of four hyperpolarization-activated pacemaker channels in cardiac and neuronal tissues. Eur. J. Biochem. 268, 1646–1652. 10.1046/j.1432-1327.2001.02036.x 11248683

[B55] Nakamura Y. Shi X. Numata T. Mori Y. Inoue R. Lossin C.. (2013). Novel HCN2 mutation contributes to febrile seizures by shifting the channel's kinetics in a temperature-dependent manner. PLoS ONE. 8:e80376. 10.1371/journal.pone.0080376 24324597 PMC3851455

[B56] Nava C. Dalle C. Rastetter A. Striano P. de Kovel C. G. Nabbout R.. (2014). De novo mutations in HCN1 cause early infantile epileptic encephalopathy. Nat. Genet. 46, 640–645. 10.1038/ng.2952 24747641

[B57] Nolan M. F. Malleret G. Dudman J. T. Buhl D. L. Santoro B. Gibbs E.. (2004). A behavioral role for dendritic integration: HCN1 channels constrain spatial memory and plasticity at inputs to distal dendrites of CA1 pyramidal neurons. Cell 119, 719–732. 10.1016/S0092-8674(04)01055-4 15550252

[B58] Nolan M. F. Malleret G. Lee K. H. Gibbs E. Dudman J. T. Santoro B.. (2003). The hyperpolarization-activated HCN1 channel is important for motor learning and neuronal integration by cerebellar Purkinje cells. Cell 115, 551–564. 10.1016/S0092-8674(03)00884-5 14651847

[B59] Nurmikko T. J. Serpell M. G. Hoggart B. Toomey P. J. Morlion B. J. Haines D. (2007). Sativex successfully treats neuropathic pain characterised by allodynia: a randomised, double-blind, placebo-controlled clinical trial. Pain. 133, 210–220. 10.1016/j.pain.2007.08.028 17997224

[B60] Ohlsson A. Lindgren J. E. Andersson S. Agurell S. Gillespie H. Hollister L. E. (1984). Single dose kinetics of cannabidiol in Man. In: Agurell S, Dewey WL, Willette RE, editors. The Cannabinoids: Chemical, Pharmacologic, Therapeutic Aspects (Academic Press), pp. 219–225. 10.1016/B978-0-12-044620-9.50020-8

[B61] Okada Y. Imendra K. G. Miyazaki T. Hotokezaka H. Fujiyama R. Zeredo J. L.. (2005). Biophysical properties of voltage-gated Na+ channels in frog parathyroid cells and their modulation by cannabinoids. J. Exp. Biol. 208, 4747–4756. 10.1242/jeb.01967 16326956

[B62] Onoda S. Masuda N. Seto T. Eguchi K. Takiguchi Y. Isobe H.. (2006). Thoracic oncology research group. phase ii trial of amrubicin for treatment of refractory or relapsed small-cell lung cancer: thoracic oncology research group study 0301. J. Clin. Oncol. 24, 5448–53. 10.1200/JCO.2006.08.4145 17135647

[B63] Orvos P. Pászti B. Topal L. Gazdag P. Prorok J. Polyák A.. (2020). The electrophysiological effect of cannabidiol on hERG current and in guinea-pig and rabbit cardiac preparations. Sci. Rep. 10:16079. 10.1038/s41598-020-73165-2 32999428 PMC7528081

[B64] Pape H. C.. (1996). Queer current and pacemaker: the hyperpolarization-activated cation current in neurons. Annu. Rev. Physiol. 58, 299–327. 10.1146/annurev.ph.58.030196.001503 8815797

[B65] Paspalas C. D. Wang M. Arnsten A. F. (2013). Constellation of HCN channels and cAMP regulating proteins in dendritic spines of the primate prefrontal cortex: potential substrate for working memory deficits in schizophrenia. Cereb. Cortex. 23, 1643–1654. 10.1093/cercor/bhs152 22693343 PMC3673177

[B66] Peng B.-W. Justice J. A. Zhang K. He X. H. Sanchez R. M. (2010). Increased basal synaptic inhibition of hippocampal area CA1 pyramidal neurons by an antiepileptic drug that enhances IH. Neuropsychopharmacology 35, 464–472. 10.1038/npp.2009.150 19776733 PMC2795055

[B67] Pike L. J. Han X. Gross R. W. (2005). Epidermal growth factor receptors are localized to lipid rafts that contain a balance of inner and outer leaflet lipids: a shotgun lipidomics study. J. Biol. Chem. 280, 26796–26804. 10.1074/jbc.M503805200 15917253 PMC1266279

[B68] Poling J. S. Rogawski M. A. Salem N. Vicini S. (1996). Anandamide, an endogenous cannabinoid, inhibits shaker-related voltage-gated K+ channels. Neuropharmacology 35, 983–991. 10.1016/0028-3908(96)00130-X 8938728

[B69] Post J. A. Verkleij A. J. Langer G. A. (1995). Organization and function of sarcolemmal phospholipids in control and ischemic/reperfused cardiomyocytes. J. Mol. Cell. Cardiol. 27, 749–760. 10.1016/0022-2828(95)90080-2 7776380

[B70] Pumroy R. A. Samanta A. Liu Y. Hughes T. E. Zhao S. Yudin Y.. (2019). Molecular mechanism of TRPV2 channel modulation by cannabidiol. Elife 8:e48792. 10.7554/eLife.48792.045 31566564 PMC6794088

[B71] Ross H. R. Napier I. Connor M. (2008). Inhibition of recombinant human T-type calcium channels by Delta9-tetrahydrocannabinol and cannabidiol. J. Biol. Chem. 283, 16124–16134. 10.1074/jbc.M707104200 18390906 PMC3259625

[B72] Russo E. B.. (2018). Cannabis therapeutics and the future of neurology. Front. Integr. Neurosci. 12:51. 10.3389/fnint.2018.00051 30405366 PMC6200872

[B73] Sait L. G. Sula A. Ghovanloo M.-R. Hollingworth D. Ruben P. C. Wallace B. A. (2020). Cannabidiol interactions with voltage-gated sodium channels. Elife 9:e58593. 10.7554/eLife.58593.sa2 33089780 PMC7641581

[B74] Santoro B. Grant S. G. Bartsch D. Kandel E. R. (1997). Interactive cloning with the SH3 domain of N-src identifies a new brain specific ion channel protein, with homology to eag and cyclic nucleotide-gated channels. Proc. Natl. Acad. Sci. U.S.A. 94, 14815–14820. 10.1073/pnas.94.26.14815 9405696 PMC25120

[B75] Santoro B. Hu L. Liu H. Saponaro A. Pian P. Piskorowski R. A.. (2011). TRIP8b regulates HCN1 channel trafficking and gating through two distinct C-terminal interaction sites. J. Neurosci. 31, 4074–4086. 10.1523/JNEUROSCI.5707-10.2011 21411649 PMC3077297

[B76] Santoro B. Liu D. T. Yao H. Bartsch D. Kandel E. R. Siegelbaum S. A.. (1998). Identification of a gene encoding a hyperpolarization-activated pacemaker channel of brain. Cell 93, 717–729. 10.1016/S0092-8674(00)81434-8 9630217

[B77] Scroggs R. S. Todorovic S. M. Anderson E. G. Fox A. P. (1994). Variation in IH, IIR, and ILEAK between acutely isolated adult rat dorsal root ganglion neurons of different size. J. Neurophysiol. 71, 271–279. 10.1152/jn.1994.71.1.271 7512627

[B78] Soliman N. Haroutounian S. Hohmann A. G. Krane E. Liao J. Macleod M.. (2021). Systematic review and meta-analysis of cannabinoids, cannabis-based medicines, and endocannabinoid system modulators tested for antinociceptive effects in animal models of injury-related or pathological persistent pain. Pain 162, S26–S44. 10.1097/j.pain.0000000000002269 33729209 PMC8216112

[B79] Stith B. J. Hall J. Ayres P. Waggoner L. Moore J. D. Shaw W. A. (2000). Quantification of major classes of *Xenopus phospholipids* by high performance liquid chromatography with evaporative light scattering detection. J. Lipid Res. 41, 1448–1454. 10.1016/S0022-2275(20)33457-X 10974052

[B80] Tang B. Sander T. Craven K. B. Hempelmann A. Escayg A. (2008). Mutation analysis of the hyperpolarization-activated cyclic nucleotide-gated channels HCN1 and HCN2 in idiopathic generalized epilepsy. Neurobiol. Dis. 29, 59–70. 10.1016/j.nbd.2007.08.006 17931874 PMC2709210

[B81] The Health Effects of Cannabis and Cannabinoids (2017). The Current State of Evidence and Recommendations for Research (Washington, DC), 85–126. 28182367

[B82] Tibbs G. R. Posson D. J. Goldstein P. A. (2016). Voltage-gated ion channels in the PNS: novel therapies for neuropathic pain? Trends Pharmacol. Sci. 37, 522–542. 10.1016/j.tips.2016.05.002 27233519

[B83] Tibbs G. R. Rowley T. J. Sanford R. L. Herold K. F. Proekt A. Hemmings H. C. Jr. (2013). HCN1 channels as targets for anesthetic and nonanesthetic propofol analogs in the amelioration of mechanical and thermal hyperalgesia in a mouse model of neuropathic pain. J. Pharmacol. Exp. Ther. 345, 363–373. 10.1124/jpet.113.203620 23549867 PMC3657108

[B84] Tu H. Deng L. Sun Q. Yao L. Han J. S. Wan Y. (2004). Hyperpolarization-activated, cyclic nucleotide-gated cation channels: roles in the differential electrophysiological properties of rat primary afferent neurons. J. Neurosci. Res. 76, 713–722. 10.1002/jnr.20109 15139030

[B85] Turcotte D. Le Dorze J. A. Esfahani F. Frost E. Gomori A. Namaka M. (2010). Examining the roles of cannabinoids in pain and other therapeutic indications: a review. Expert Opin. Pharmacother. 11, 17–31. 10.1517/14656560903413534 20001426

[B86] Villiere V. McLachlan E. M. (1996). Electrophysiological properties of neurons in intact rat dorsal root ganglia classified by conduction velocity and action potential duration. J. Neurophysiol. 76, 1924–1941. 10.1152/jn.1996.76.3.1924 8890304

[B87] Williamson E. M.. (2004). The Medicinal Uses of Cannabis Cannabinoids. Edited by Guy GW, Whittle BA, Robson PJ. Pharmaceutical Press: London, 2004. *Pages: 448. ISBN: 0-85369-517-2*. *Price £39.95*. Human Psychopharmacology: Clinical and Experimental. 19:589. 10.1002/hup.631

[B88] Willis W. D.. (1997). Central sensitization following intradermal injection of capsaicin. Behav. Brain Sci. 20:471. 10.1017/S0140525X9758149016549018

[B89] Xiao Y. F. Chandler N. Dobrzynski H. Richardson E. S. Tenbroek E. M. Wilhelm J. J.. (2010). Hysteresis in human HCN4 channels: a crucial feature potentially affecting sinoatrial node pacemaking. Sheng Li Xue Bao 62, 1–13. 20179882

[B90] Xiong W. Cui T. Cheng K. Yang F. Chen S. R. Willenbring D.. (2012). Cannabinoids suppress inflammatory and neuropathic pain by targeting alpha3 glycine receptors. J. Exp. Med. 209, 1121–1134. 10.1084/jem.20120242 22585736 PMC3371734

[B91] Yagi J. Sumino R. (1998). Inhibition of a hyperpolarization-activated current by clonidine in rat dorsal root ganglion neurons. J. Neurophysiol. 80, 1094–1104. 10.1152/jn.1998.80.3.1094 9744924

[B92] Yan J. Y. Sun R. Q. Hughes M. G. McAdoo D. J. Willis W. D. (2006). Intradermal injection of capsaicin induces acute substance P release from rat spinal cord dorsal horn. Neurosci. Lett. 410, 183–186. 10.1016/j.neulet.2006.09.072 17101224

[B93] Yao H. Donnelly D. F. Ma C. LaMotte R. H. (2003). Upregulation of the hyperpolarization-activated cation current after chronic compression of the dorsal root ganglion. J. Neurosci. 23, 2069–2074. 10.1523/JNEUROSCI.23-06-02069.2003 12657665 PMC6742022

[B94] Yi F. Danko T. Botelho S. C. Patzke C. Pak C. Wernig M.. (2016). Autism-associated SHANK3 haploinsufficiency causes Ih channelopathy in human neurons. Science. 352:aaf2669. 10.1126/science.aaf2669 26966193 PMC4901875

[B95] Yoshida K. Nagatoishi S. Kuroda D. Suzuki N. Murata T. Tsumoto K. (2019). Phospholipid membrane fluidity alters ligand binding activity of a G Protein-Coupled Receptor by shifting the conformational equilibrium. Biochemistry 58, 504–508. 10.1021/acs.biochem.8b01194 30618239

[B96] Zar J. H.. (1984). Biostatistical Analysis. Englewood Cliffs, NJ: Prentice-Hall.

