# Supplementary material for: Direct Regulation of Hyperpolarization-Activated Cyclic-Nucleotide Gated (HCN1) Channels by Cannabinoids
Source: Front Mol Neurosci. 2022 Apr 6;15:848540. doi: 10.3389/fnmol.2022.848540 (PMC9019169; doi:10.3389/fnmol.2022.848540)
Supplement: Supplementary file 1 [file Data_Sheet_1.docx]

Direct regulation of HCN1 channels by cannabinoids

Sultan Mayar, Mina Memapoor-Yazdi, Ahmad Makky, Nazzareno D’Avanzo

^1^Université de Montréal, Département de pharmacologie et physiologie, Montréal, Québec, Canada

*** Correspondence:**Nazzareno D’Avanzo nazzareno.d.avanzo@umontreal.ca

Supplementary Material

# Supplementary Figures and Tables

**
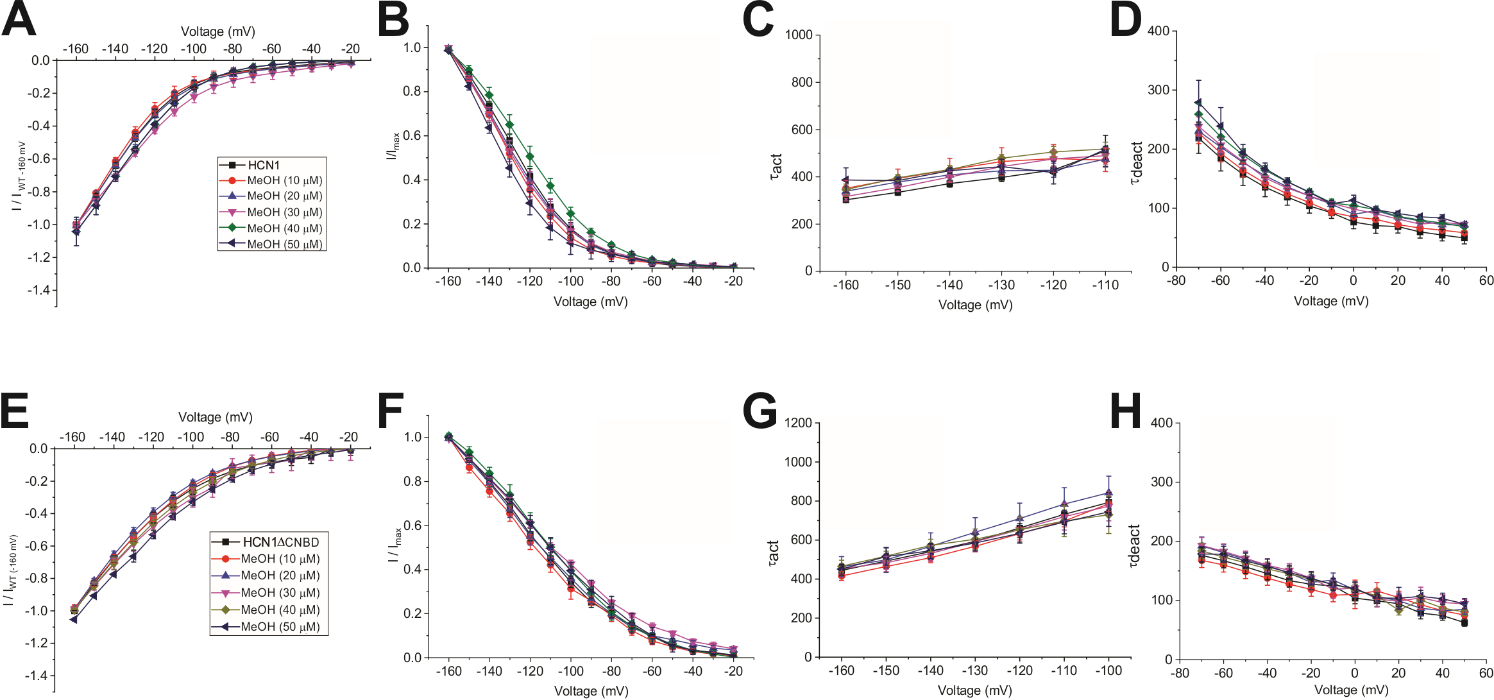
**

**Supplementary Figure 1.** **Regulation of full-length HCN1 and HCN1-ΔCNBD by methanol (MeOH). (A)** Current-voltage (I/V) relationship of full-length HCN1 in presence of MeOH normalized to maximal current (I_WT (-160 mV)_). MeOH quantities used were equivalent to the amounts used as a vehicle during the addition of the concentration of cannabinoids shown in brackets (4 < n < 10 per condition; 0.45 < P < 0.87 G_max_ (slope between -120mV and -160mV)). **(B)** Steady-state activation of full-length HCN1 in presence of MeOH. (P = 0.14 for V_1/2_). **(C)** Activation time constant (τ) kinetics of full-length HCN1 in presence of MeOH. (0.12 < P < 0.87). **(D)** Deactivation time constant (τ) kinetics of full-length HCN1 in presence of MeOH. (n = 3-4 per condition; 0.23 < P < 0.83). (**E)** Current-voltage (I/V) relationship of HCN1-ΔCNBD in presence of MeOH normalized to maximal current (I_WT (-160 mV)_). (4 < n < 11 per condition; 0.16 < P < 0.93) **(F)** Steady-state activation of HCN1-ΔCNBD in presence of MeOH. (P = 0.89 for V_1/2_) **(G)** Activation time constant (τ) kinetics for of HCN1-ΔCNBD in presence of MeOH. **(H)** Deactivation time constant (τ) kinetics of HCN1-ΔCNBD in presence of MeOH. (4 < n < 6 per condition; 0.23 < P < 0.63)


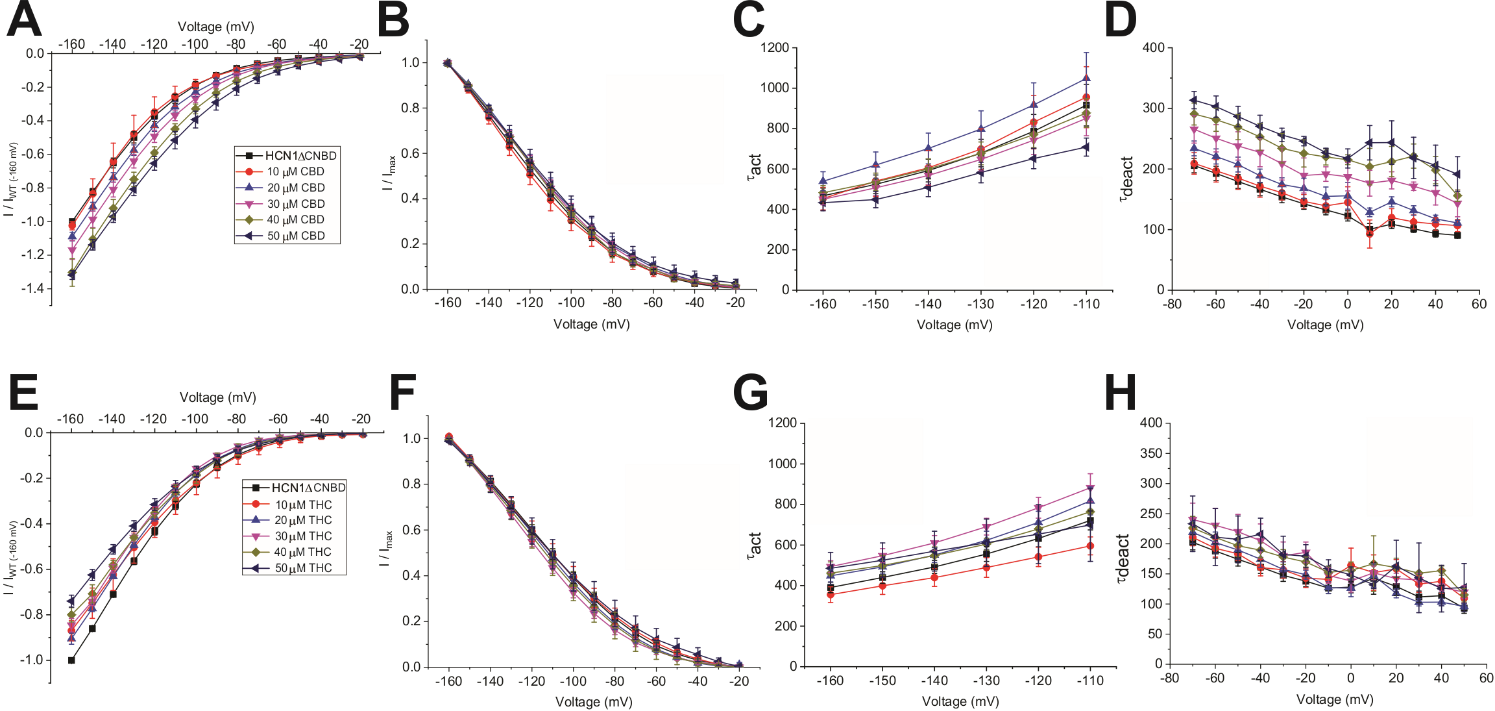


**Supplementary Figure 2.** **Regulation of HCN1-ΔCNBD by cannabidiol (CBD) and Δ^9^-tetrahydrocannabidiol (THC). (A)** Current-voltage (I/V) relationship in presence of CBD normalized to maximal current (I_WT (-160 mV)_) (4 < n < 13 per condition; P < 0.05 for 20 - 50 μM). **(B)** Steady state activation in presence of CBD (P = 0.63 for V_1/2_). **(C)** Activation time constant (τ) kinetics in presence of CBD (0.11 < P < 0.46) **(D)** Deactivation time constant (τ) kinetics in presence of CBD (4 < n < 10 per condition; P < 0.05 for 20-50 μM). **(E)** Current-voltage (I/V) relationship in presence of THC normalized to maximal current (I_WT (-160 mV)_) (4 < n < 12 per condition; P < 0.05 for 10 - 50 μM). **(F)** Steady state activation in presence of THC. (P = 0.34 for V_1/2_). **(G)** Activation time constant (τ) kinetics in presence of THC (0.12 < P < 0.46). **(H)** Deactivation time constant (τ) kinetics in presence of THC (4 < n < 11 per condition; 0.23 < P < 0.84).

**
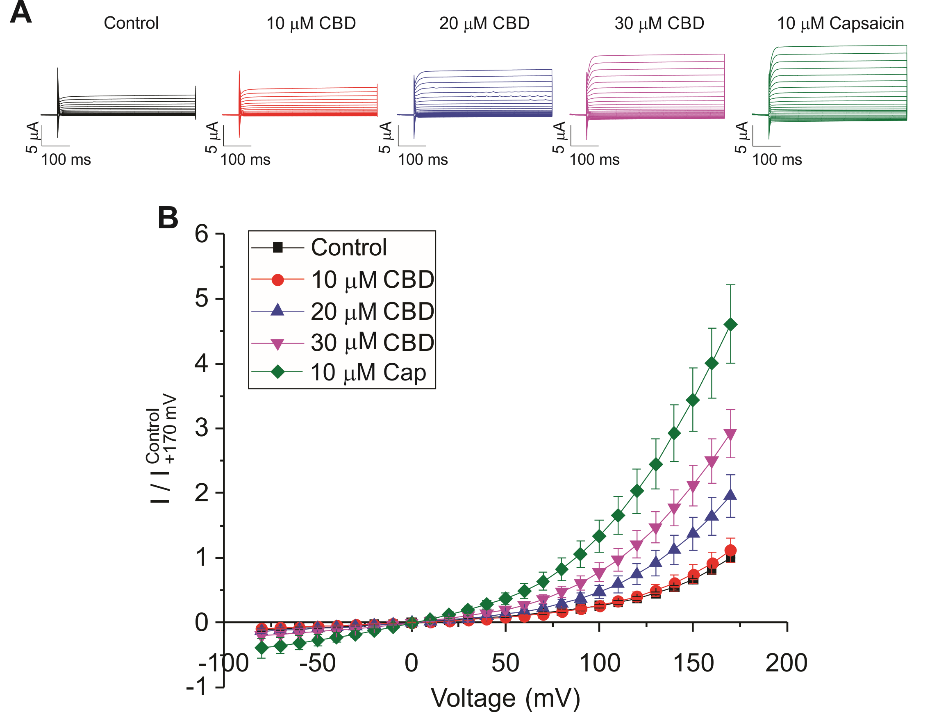
**

Supplementary Figure 3. Activation of TRPV1 by CBD. (A) Representative traces from a paired experiment following the addition of increasing concentrations of CBD to oocytes expressing full-length rTRPV1. Membrane voltage was stepped from a holding potential of 0 mV to –80 to 170 mV. 10 μM Capsaicin was added at the end of each experiment. (B) Normalized current voltage relationship (I/I_(Control +170 mV)_) after addition of increasing concentrations of CBD shown (n=8; P < 0.05 for Gmax (slope between +120 and +170 mV) of 20 CBD, 30 μM CBD and Cap vs. Control).

Supplemental Table 1: Steady-state activation data for HCN1 in presence of cannabinoids

| **Condition** | **V½ (mV)** | **k** |
| --- | --- | --- |
| HCN1 Control | -125.2 ± 1.7 | 18.5 ± 0.3 |
| HCN1 + MeOH (10 µM) | -128.8 ± 2.3 | 17.6 ± 0.5 |
| HCN1 + MeOH (20 µM) | -127.1 ± 2.1 | 17.7 ± 0.7 |
| HCN1 + MeOH (30 µM) | -128.1 ± 1.2 | 18.5 ± 0.5 |
| HCN1 + MeOH (40 µM) | -119.7 ± 4.2 | 19.6 ± 1.0 |
| HCN1 + MeOH (50 µM) | -132.7 ± 6.6 | 16.9 ± 1.3 |
| HCN1 Control | -122.2 ± 0.4 | 21.1 ± 0.2 |
| HCN1 + 10 µM CBD | -122.8 ± 0.7 | 20.6 ± 0.5 |
| HCN1 + 20 µM CBD | -125.6 ± 2.8 | 17.6 ± 0.8 |
| HCN1 + 30 µM CBD | -118.9 ± 1.9 | 24.0 ± 0.3 |
| HCN1 + 40 µM CBD | -119.6 ± 1.5 | 22.9 ± 0.3 |
| HCN1 + 50 µM CBD | -116.8 ± 2.4 | 15.1 ± 1.9 |
| HCN1 Control | -129.8 ± 2.7 | 17.0 ± 0.3 |
| HCN1 + 10 µM THC | -129.2 ± 0.7 | 16.6 ± 0.5 |
| HCN1 + 20 µM THC | -125.9 ± 4.2 | 14.7 ± 0.6 |
| HCN1 + 30 µM THC | -129.3 ± 3.4 | 20.6 ± 1.3 |
| HCN1 + 40 µM THC | -131.8 ± 4.9 | 17.6 ± 1.0 |
| HCN1 + 50 µM THC | -132.8 ± 5.3 | 17.9 ± 1.1 |

Supplemental Table 2: Steady-state activation data for HCN1-ΔCNBD in presence of cannabinoids

| **Condition** | **V½ (mV)** | **k** |
| --- | --- | --- |
| HCN1 Control | -115.9 ± 2.1 | 22.6 ± 0.5 |
| HCN1 + MeOH (10 µM) | -118.3 ± 3.7 | 23.4 ± 0.6 |
| HCN1 + MeOH (20 µM) | -115.2 ± 2.3 | 22.9 ± 0.8 |
| HCN1 + MeOH (30 µM) | -110.2 ± 4.4 | 23.1 ± 0.4 |
| HCN1 + MeOH (40 µM) | -111.5 ± 2.7 | 22.8 ± 0.3 |
| HCN1 + MeOH (50 µM) | -111.8 ± 3.2 | 22.9 ± 0.3 |
| HCN1 Control | -117.8 ± 3.6 | 24.4 ± 0.3 |
| HCN1 + 10 µM CBD | -119.8 ± 4.5 | 22.9 ± 0.6 |
| HCN1 + 20 µM CBD | -115.3 ± 2.5 | 23.5 ± 0.8 |
| HCN1 + 30 µM CBD | -116.9 ± 1.1 | 29.9 ± 1.1 |
| HCN1 + 40 µM CBD | -114.3 ± 1.8 | 25.3 ± 2.3 |
| HCN1 + 50 µM CBD | -113.8 ± 2.4 | 25.1 ± 2.2 |
| HCN1 Control | -113.9 ± 5.4 | 28.6 ± 0.6 |
| HCN1 + 10 µM THC | -114.3 ± 4.8 | 28.5 ± 0.5 |
| HCN1 + 20 µM THC | -115.2 ± 1.3 | 26.9 ± 1.2 |
| HCN1 + 30 µM THC | -116.2 ± 3.4 | 26.2 ± 0.4 |
| HCN1 + 40 µM THC | -115.2 ± 2.7 | 27.6 ± 1.0 |
| HCN1 + 50 µM THC | -112.8 ± 3.2 | 25.3 ± 0.6 |

Supplemental Table 3: Steady-state activation data for HCN1 in presence of TX-100

| **Condition** | **V½ (mV)** | **k** |
| --- | --- | --- |
| HCN1 Control | -125.6 ± 1.6 | 19.4 ± 0.2 |
| HCN1 15 µM TX-100 | -134.7 ± 1.8 * | 19.2 ± 0.3 |
| HCN1 25 µM TX-100 | -134.1 ± 2.3 * | 21.1 ± 0.3 |
| HCN1 30 µM TX-100 | -133.6 ± 2.1 * | 18.5 ± 0.6 |
| HCN1 45 µM TX-100 | -140.3 ± 0.7 * | 16.6 ± 0.2 |

(* P < 0.05 vs. Control)
